# The role of multimodal cardiac imaging in managing electrical storms in severe heart calcifications: a case report

**DOI:** 10.1093/ehjcr/ytaf068

**Published:** 2025-02-14

**Authors:** Adrienne Lejeune, Dominique Blommaert, Bernard Cosyns, Julien Higny, Maria-Luiza Luchian

**Affiliations:** Department of Cardiology, Université catholique de Louvain (UCL), CHU UCL Namur Site Godinne, Av. Dr. G. Thérasse, 1, Yvoir 5530, Belgium; Department of Cardiology, Université catholique de Louvain (UCL), CHU UCL Namur Site Godinne, Av. Dr. G. Thérasse, 1, Yvoir 5530, Belgium; Department of Cardiology, Centrum voor Hart-en Vaatziekten, Universitair Ziekenhuis Brussel, Vrije Universiteit Brussel (VUB), Laarbeeklaan 101, Brussels 1090, Belgium; Department of Cardiology, Université catholique de Louvain (UCL), CHU UCL Namur Site Godinne, Av. Dr. G. Thérasse, 1, Yvoir 5530, Belgium; Department of Cardiology, Université catholique de Louvain (UCL), CHU UCL Namur Site Godinne, Av. Dr. G. Thérasse, 1, Yvoir 5530, Belgium

**Keywords:** Heart calcification, Myocardial calcification, Electrical storm, Ventricular tachycardia, Defibrillator, Case report

## Abstract

**Background:**

Intramyocardial calcifications are rare and associated with conditions such as myocardial infarction, rheumatic heart disease, and calcium metabolism disorders. These calcifications carry significant prognostic value, often leading to severe complications like ventricular arrhythmias, increased morbidity, and mortality. They can pose challenges for treatment, especially when ablation is ineffective due to the calcifications acting as physical barriers.

**Case summary:**

A 43-year-old male with a history of extensive myocardial calcifications and recurrent ventricular tachycardia (VT) presented with a prolonged electrical storm, despite having an implantable cardioverter-defibrillator (ICD). Multiple ICD shocks and overdrive pacing temporarily restored sinus rhythm, but VT recurred. Initial management with amiodarone and electrical cardioversions failed to control the arrhythmias. The patient required sedation and intubation for 36 h. High-dose amiodarone and general anaesthesia eventually stabilized the arrhythmia. Post-sedation, the patient was discharged with oral amiodarone and bisoprolol, without further arrhythmia.

**Discussion:**

This case underscores the challenges in managing electrical storms in patients with extensive intramyocardial calcifications, which hinder ablation procedure and contribute to persistent arrhythmias. Effective management of life-threatening arrhythmias in these patients requires a comprehensive approach, including multimodality cardiac imaging, collaborative decision-making by a multidisciplinary team, advanced antiarrhythmic therapy, and sedation when necessary.

**Learning points**

- The detection of cardiac calcifications can be performed by various imaging techniques, with echocardiography playing a pivotal role.
- Substantial myocardial calcifications should prompt thorough investigation to outline the underlying cause, enabling risk assessment and tailored management, given that certain causes may be reversible.

## Introduction

Intramyocardial calcifications are a rare phenomenon associated with several conditions, carrying prognostic significance. Cardiac imaging typically identifies calcium deposition in the valvular apparatus, coronary arteries, and pericardium. Intramyocardial calcifications are uncommon and usually linked to previous cardiac injuries, including myocardial infarction, rheumatic heart disease, myocarditis, and infections such as tuberculosis or sepsis.^1^ Additionally, calcium metabolism disorders, hyperparathyroidism, or chronic renal failure may lead to calcium depositions in the heart tissues, known as metastatic calcifications^1,2^ (*Table 1*). The presence of myocardial calcifications is often linked to heightened morbidity and mortality rates. The complete calcification of the left atrium (LA), known as ‘coconut heart’, has been described in rheumatic heart disease, whereas the presence of extensive myocardial calcifications is sometimes referred to as the ‘porcelain heart’.^3^

**Table 1:** Differential diagnosis of heart calcifications

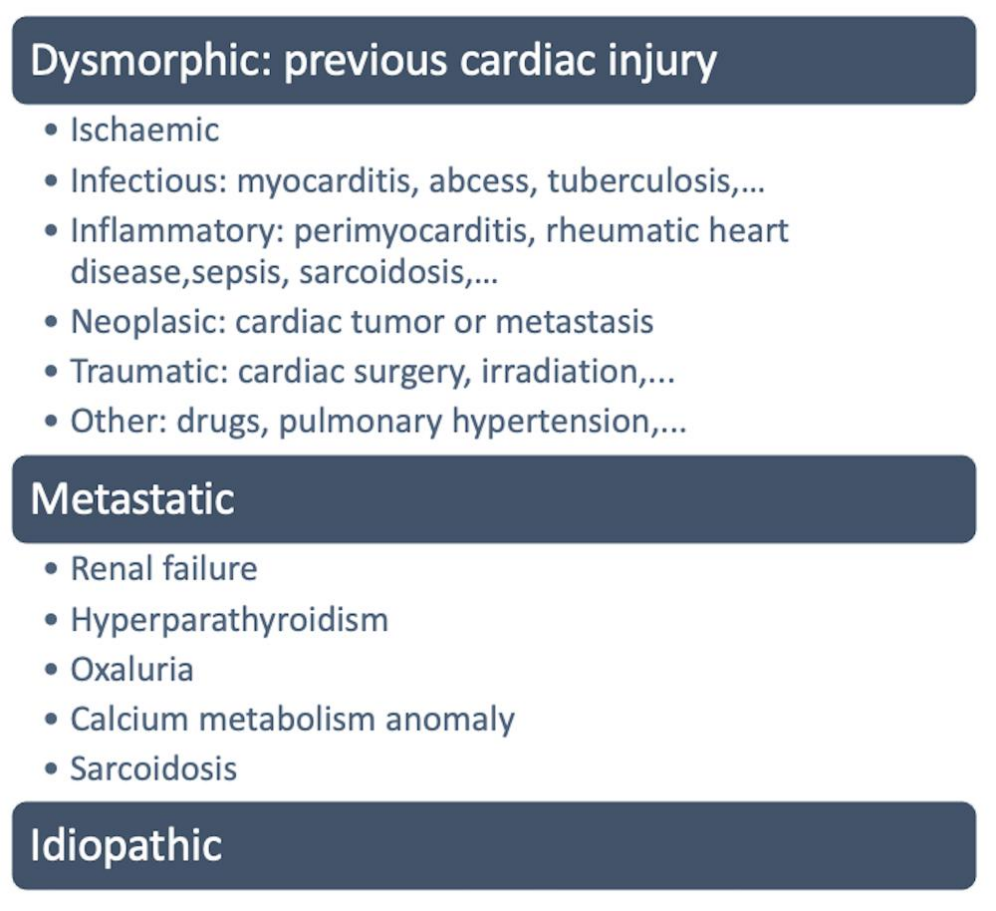

We present a case report about cardiac calcifications with multiple complications.

## Summary figure

**Figure:**
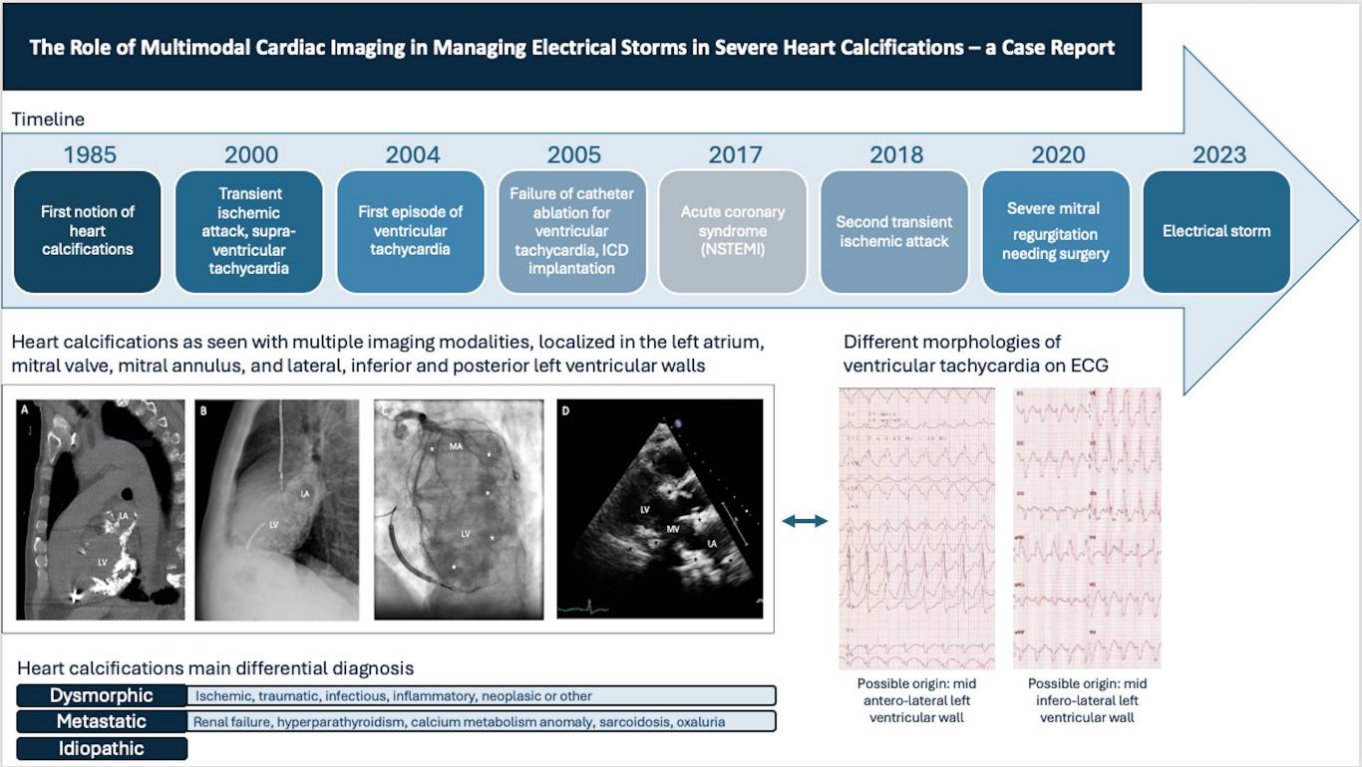
(*A*) Non-contrast computed tomography (CT) showing extensive amorphous confluent calcifications in the left ventricular (LV) wall, sparing the septum, and extending to the mitral-aortic annulus and the LA. (*B*) Chest radiography showing calcifications in the LV wall, extending to the mitral-aortic annulus and the LA. (*C*) Calcifications (marked with white stars) in the LV myocardium and the mitral annulus (MA) as seen on fluoroscopy during the coronary angiography. (*D*) Transthoracic echocardiographic parasternal long-axis view showing the extensive calcifications (marked with black stars) of the mitral valve (MV), extending to the mitral-aortic annulus, with intramyocardial calcification of the posterobasal and basal anteroseptal LV walls, and significant calcification of the LA. ICD indicates implantable cardioverter-defibrillator and NSTEMI myocardial infarction without ST elevation.

## Case presentation

**Figure 1:**
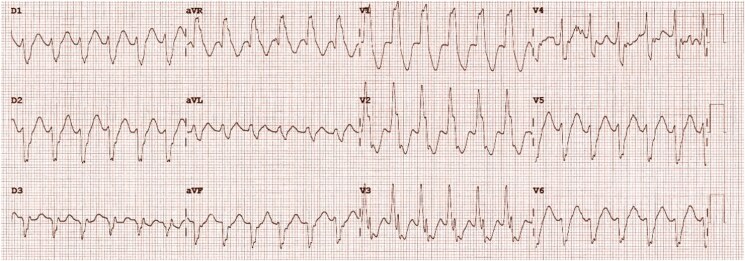
Electrocardiogram at admission.

In 2023, a 43-year-old white Western European male was admitted to the emergency department with an acute onset of persistent, regular palpitations. The electrocardiogram (ECG) indicated regular tachycardia with a wide QRS complex (*Figure 1*), consistent with ventricular tachycardia (VT). The patient had experienced the first VT episode in 2004, followed by another in 2005 while biking. Before that, his medical history consisted only of migraines and a transient ischaemic attack in 2000. In 2004, a transthoracic echocardiography (TTE) showed preserved LV function without significant abnormalities, except for calcifications of the MV, MA, and LA. A history of myocardial calcifications dating back to 1985 was noted. A thorough evaluation was conducted to rule out causes of severe calcification, including endomyocardial fibrosis and calcific constrictive pericarditis. Cardiac magnetic resonance and various blood tests, such as an intradermal tuberculin test, 24-h urinary calcium measurement, whole-body PET-scan, wrist X-ray, and a vitamin D (1.25) assay, returned negative. A previous rheumatic heart disease was suspected but never confirmed. A catheter ablation procedure was attempted but was unsuccessful. During the procedure, VT of three distinct morphologies was induced. Neither endocavitary nor epicardial radiofrequency applications produced any effect. A defibrillator was thus implanted for secondary prevention. The VT episodes persisted but were well controlled initially with sotalol, later switched to amiodarone, which was discontinued in 2021 due to severe hepatitis. Bisoprolol was added to the patient’s treatment.

In addition to recurrent VT, the patient experienced several other cardiovascular complications. In 2017, he presented with an acute coronary syndrome without ST elevation, due to occlusion of the distal circumflex artery. It was treated medically, because of the artery’s small calibre. In 2018, he suffered from a transient ischaemic attack. By 2020, he required surgical valvuloplasty for severe MV regurgitation caused by posterior prolapse, accompanied by LV dilation and severe pulmonary hypertension.

**Figure 2:**
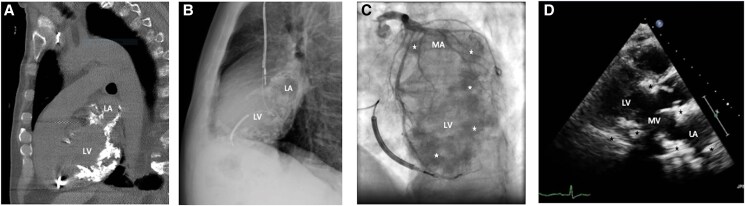
Multimodal cardiac imaging for the detection of heart calcifications. (*A*) Non-contrast computed tomography showing extensive amorphous confluent calcifications in the left ventricular wall, sparing the septum, and extending to the mitral-aortic annulus and the left atrium. (*B*) Chest radiography showing calcifications in the left ventricular wall, extending to the mitral-aortic annulus and the left atrium. (*C*) Calcifications (marked with stars) in the left ventricular myocardium and the mitral annulus as seen on fluoroscopy during the coronary angiography. (*D*) Transthoracic echocardiographic parasternal long-axis view showing the extensive calcifications (marked with black stars) of the mitral valve, extending to the mitral-aortic annulus, with intramyocardial calcification of the posterobasal and basal anteroseptal left ventricular walls, and significant calcification of the left atrium.

**Figure 3:**
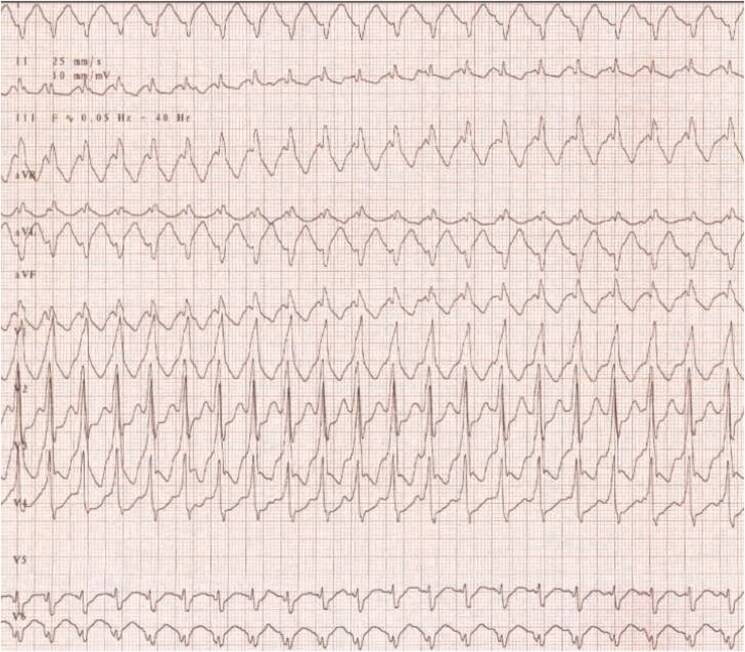
Electrocardiogram of ventricular tachycardia with a different morphology.

Upon arrival at the emergency department in 2023, the patient had been experiencing VT for 4 h, with episodes of both slow VTs (110–115 b.p.m.) and faster VTs (147–160 b.p.m.). Blood tests and clinical examination were unremarkable, showing good haemodynamic tolerance, no cardiac murmur, and no signs of pulmonary or systemic congestion. His ICD had attempted overdrive pacing several times, sometimes successfully, and had delivered a total of five electrical shocks, with brief returns to sinus rhythm followed by early VT recurrence. Despite the administration of a loading dose of amiodarone, VT kept recurring, and another shock was delivered by the ICD. Consequently, sedation and intubation were employed to manage the storm. Intravenous amiodarone was continued before transitioning to oral medication. There was a temporary requirement for low-dose vasopressor support, which was quickly weaned off after sedation was stopped. The patient’s condition improved, allowing for gradual withdrawal of anaesthetic medications and a switch to benzodiazepines. Sedation was lifted after 36 h, with no recurrence of arrhythmia. The patient remained free of arrhythmias and was subsequently discharged from the hospital on a regimen of amiodarone (200 mg once daily) and bisoprolol (2.5 mg twice daily). An ischaemic origin was excluded. A potential septic factor was suspected, prompting a complete evaluation for infectious causes. The TTE revealed a preserved LV ejection fraction and extensive calcification of the MV, extending to the mitral-aortic annulus, with intramyocardial calcification of the posterobasal, laterobasal, and inferobasal walls, and significant calcification of the LA (*Figure 2*; Supplementary material online, *Videos S1–S3*). Non-contrast CT showed extensive amorphous confluent calcifications in the LV wall, sparing the septum, and extending to the mitral-aortic annulus and both coronary arteries (*Figure 2*). The calcifications could also be seen on the fluoroscopy performed during the coronary angiography (*Figure 2*). The calcifications’ localizations were consistent with various VT morphologies on ECG. One ECG indicated that the VT may originate from the mid antero-lateral regions of the LV (*Figure 3*), while another suggested a mid infero-lateral origin (*Figure 1*).

At the last follow-up visit, one and a half years after this episode, the patient remained free of any arrhythmia episodes and continued to be treated with amiodarone without any significant adverse effects.

## Discussion

There are three different patterns of myocardial calcifications: dystrophic, metastatic, or idiopathic.^1^ In the first scenario, the calcium deposition is exacerbated by the ischaemia-induced microenvironment, characterized by relative alkalinity, reduced calcium solubility, and carbon dioxide production. One study showed dystrophic calcifications in 8% of myocardial infarctions older than 6 years.^1,4^ In this case report, the first mention of intramyocardial calcifications was before experiencing a cardiac ischaemic event. Conversely, metastatic calcifications are related to a systemic process, centred around the calcium metabolism, with hypercalcaemia and/or abnormalities of calcium homeostasis,^1,2^ which was excluded. Any abnormality in calcium metabolism can lead to metastatic calcification, including conditions such as renal failure, bone destruction or increased bone turnover, hyperparathyroidism, and vitamin D-related disorders, which was excluded in our case.^5,6^ Presumably, idiopathic calcifications are a form of dystrophic or metastatic calcifications resulting from various past pathological conditions, sometimes underdiagnosed. Several reports describe the presence of intramyocardial and pericarditic causes, related to extracardiac conditions such as tuberculosis, cardiac sarcoidosis, irradiation, severe viral infections, or autoimmune diseases, that may further lead to constriction, altering the LV function.^1,6^

Intramyocardial calcification warrants careful attention, as its severity surpasses initial impressions. It frequently precipitates heart failure in chronic haemodialysis patients and poses a risk of sudden cardiac death.^7^ These calcifications often coincide with focal wall motion abnormalities, as a substrate for life-threatening arrhythmias. In the present case report, the suspected origins of VT on ECG corresponded with the localizations of the calcifications. They may delineate areas of abnormal excitability, facilitating re-entry circuits, particularly in cases of extensive left atrial calcifications.^7,8^ This further may impede effective ablation procedures, serving as a physical barrier. In this case, both endocavitary and epicardial ablation were unsuccessful, likely due to the endomyocardial origin of the VT being inaccessible because of calcifications. The patient experienced both ventricular and supra-VT, with arrhythmia control strategies becoming ineffective over time.

## Conclusion

This case perfectly illustrates the myriad complications associated with cardiac calcifications, including ventricular and supra-ventricular arrhythmias. Cardiac calcifications can be detected through various imaging techniques, particularly echocardiography. While often incidental findings, their origin often remains uncertain. Substantial myocardial calcifications warrant comprehensive investigation to determine underlying cause, enabling risk assessment and tailored management, especially when certain causes are reversible.

## Lead author biography


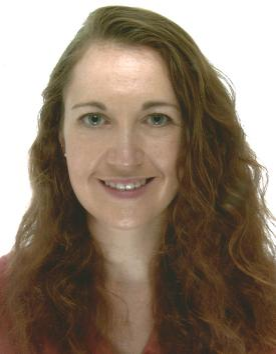


Adrienne Lejeune is an intern in cardiology. She graduated in medicine in 2018 in the Catholic University of Louvain (UCL). She has been working in Belgium as an intern since then, first in internal medicine and then in cardiology since 2021.

## Supplementary Material

ytaf068_Supplementary_Data

## Data Availability

The main data underlying this article are available in the article and its online Supplementary material. Additional data will be shared on reasonable request to the corresponding author.

## Supplementary material

**Consent:** The patient gave consent to appear in the publication in accordance with the COPE guidelines.

**Funding:** None to declare.
